# Supplementary material for: Single-cell transcriptome analysis reveals cellular reprogramming and changes of immune cell subsets following tetramethylpyrazine treatment in LPS-induced acute lung injury
Source: PeerJ. 2025 Jan 13;13:e18772. doi: 10.7717/peerj.18772 (PMC11737342; doi:10.7717/peerj.18772)
Supplement: Supplemental Information 2 [file peerj-13-18772-s002.docx]

**Supplementary Figures**


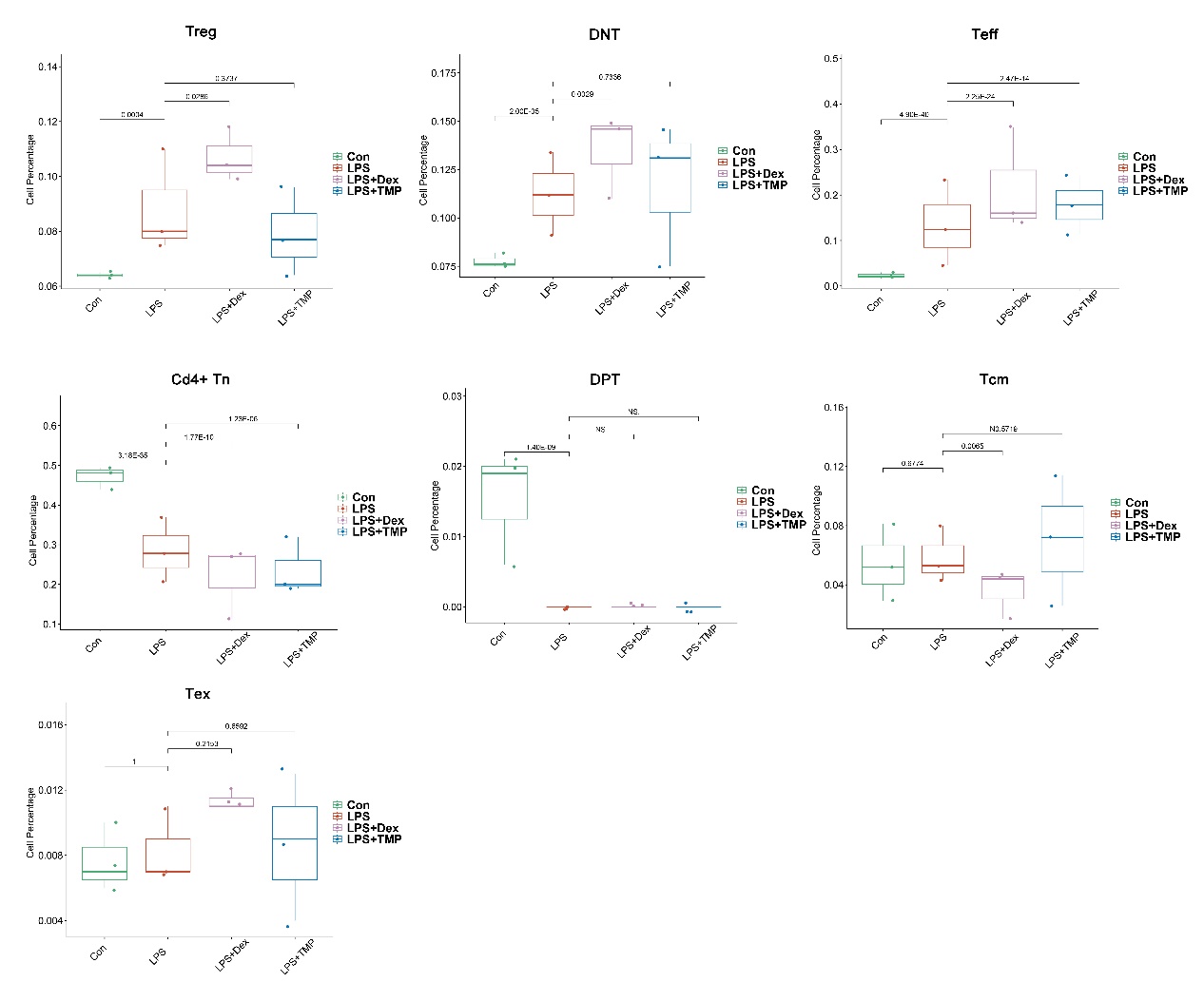


**Figure S1**

The cell percentage of the T cell sub-clusters as presented among the different groups. The results showed that LPS increased the cell numbers of Double Negative T cells (DNT), Effector T cells (Teff) and Regulatory T cells (Treg), whereas there were no significant inhibitory effects of TMP observed in the LPS-induced cell number changes in these cell subsets. Additionally, LPS did not significantly promote other cell subpopulations.


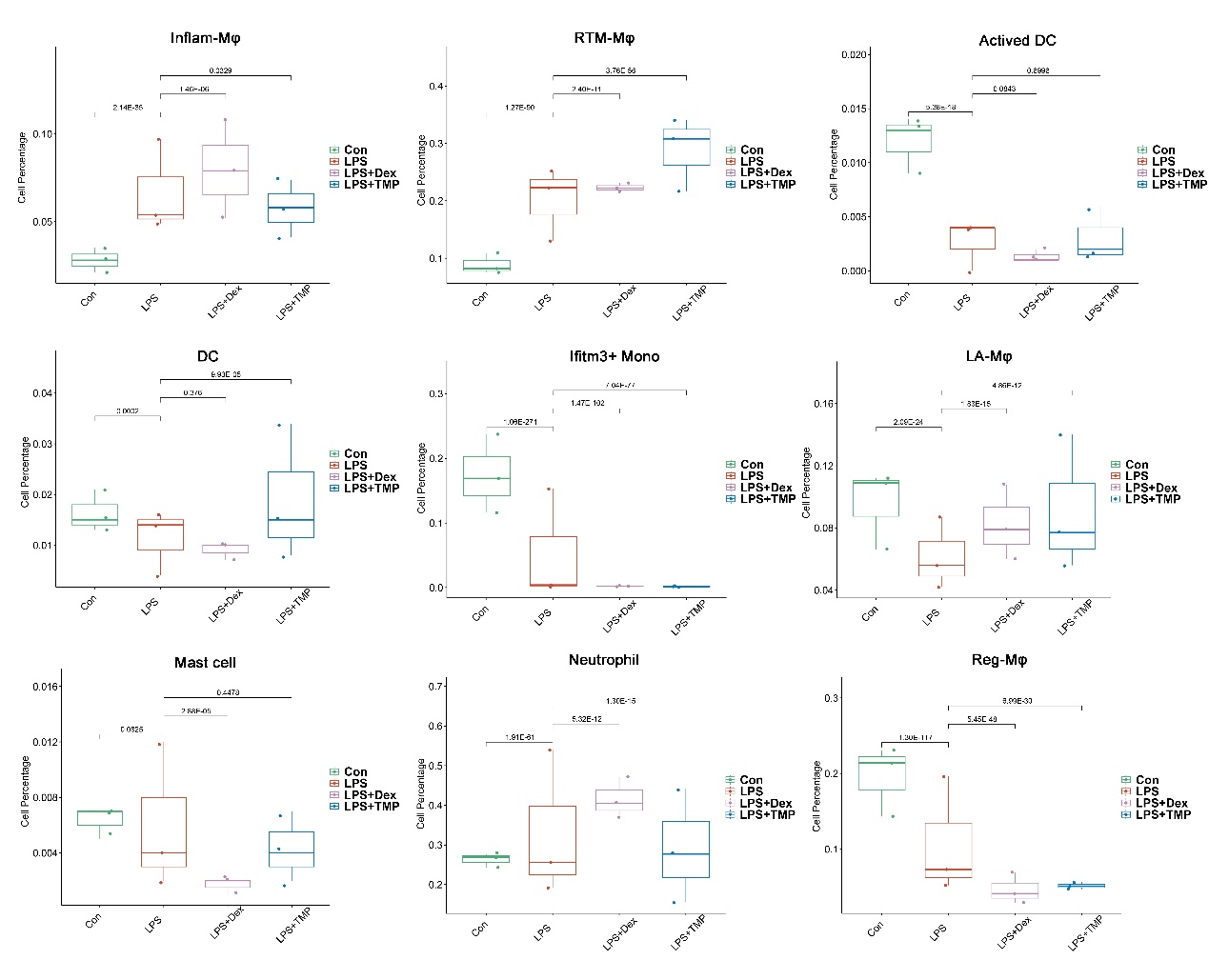


**Figure S2**

The cell percentage of the myeloid cell sub-clusters as presented among the different groups. We found that LPS led to an increased percentage of inflammatory Macrophages (Inflam-Mφ) and resident Tissue Macrophages (RTM-Mφ) cells, whereas there were no significant inhibitory effects of TMP observed in these two cell subsets. Additionally, LPS did not significantly promote other cell subpopulations.


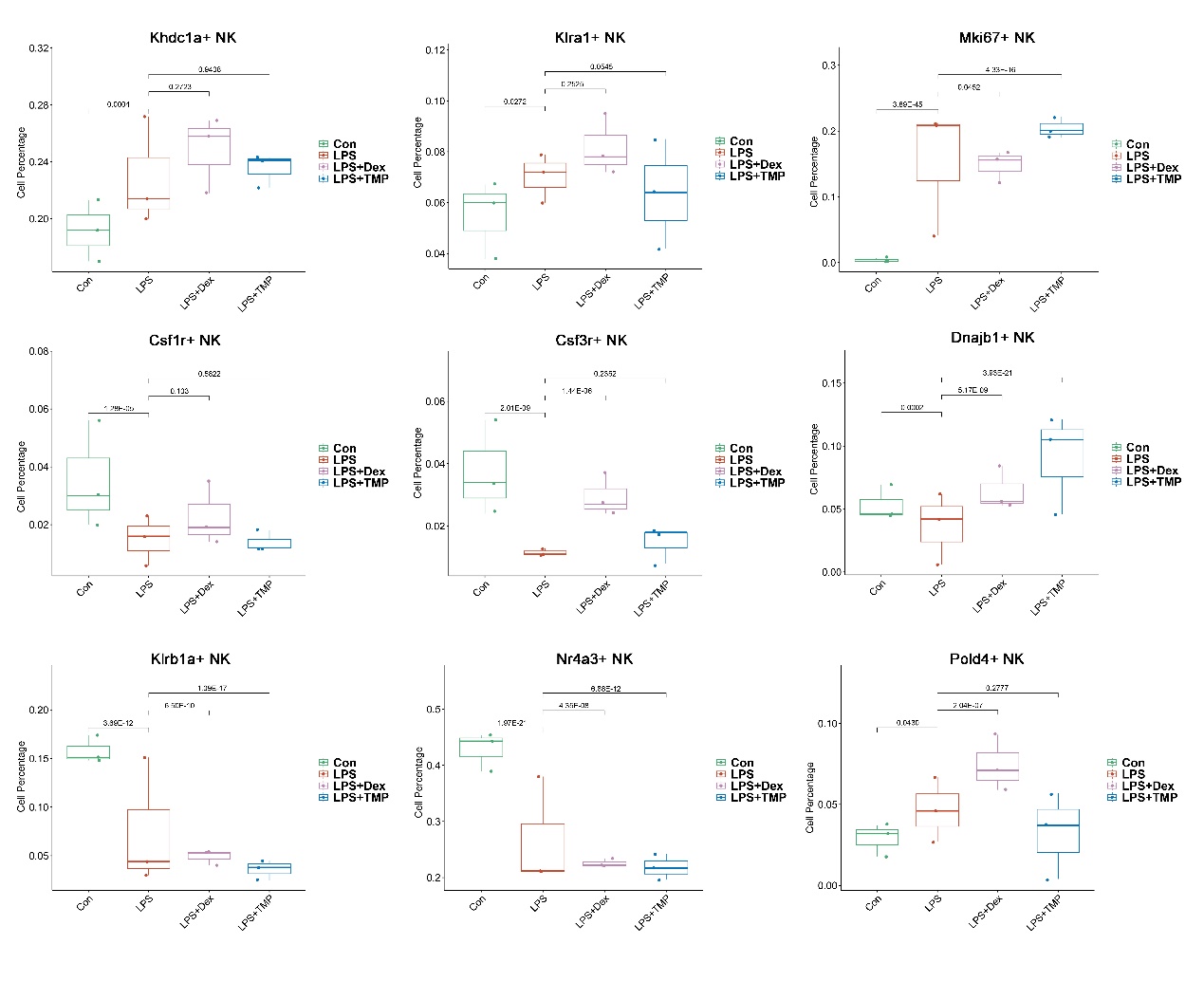


**Figure S3**

The results showed that LPS induced an increased number of Khdc1a-positive, Klra1-positive and Mki67-positive NK cells, while treatment with either Dex or TMP only slightly inhibited the effects of LPS in MKI67-positive NK cells. Additionally, LPS slightly increased the number of Pold4-positive NK cells and did not significantly promote other cell subpopulations.


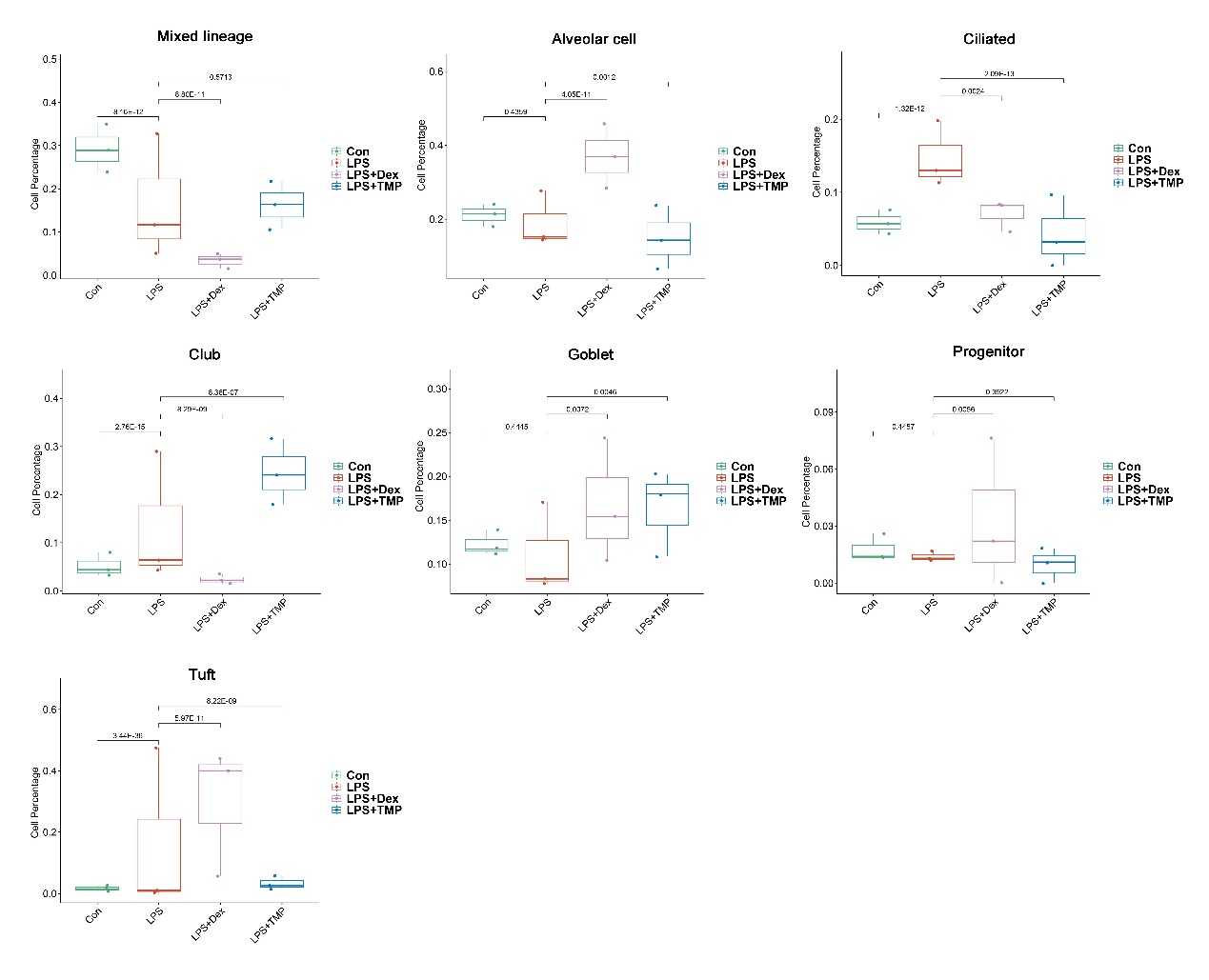


**Figure S4**

The cell percentage of epithelial cell sub-clusters as presented among the different groups. We found that LPS decreased the cell number of mixed lineage cell subsets and treatment with Dex led to further reduction in cell numbers, but no significant protective effects were found following the treatment with TMP when compared with the LPS group. Additionally, LPS did not significantly decrease other cell subpopulations.


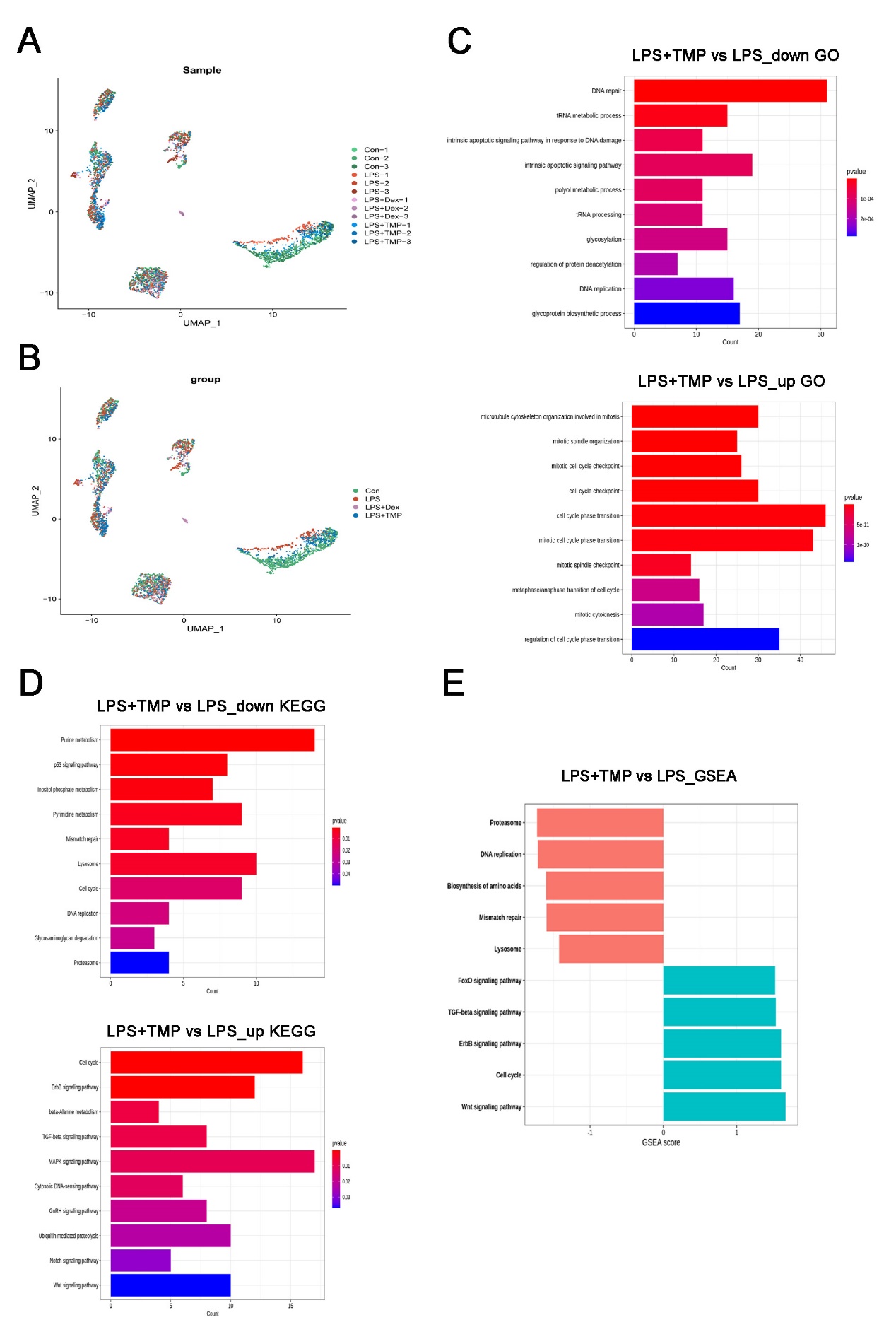


**Figure S5**

A and B: The cell subsets of epithelial cell, which were generally present in different samples (A) and groups (B). C and D: Enrichment analysis of the differentially expressed genes in basal cells obtained after treatment with LPS and LPS+TMP using GO (C) and KEGG (D) analysis, respectively. E: GSEA analysis of the differentially expressed genes in basal cells.


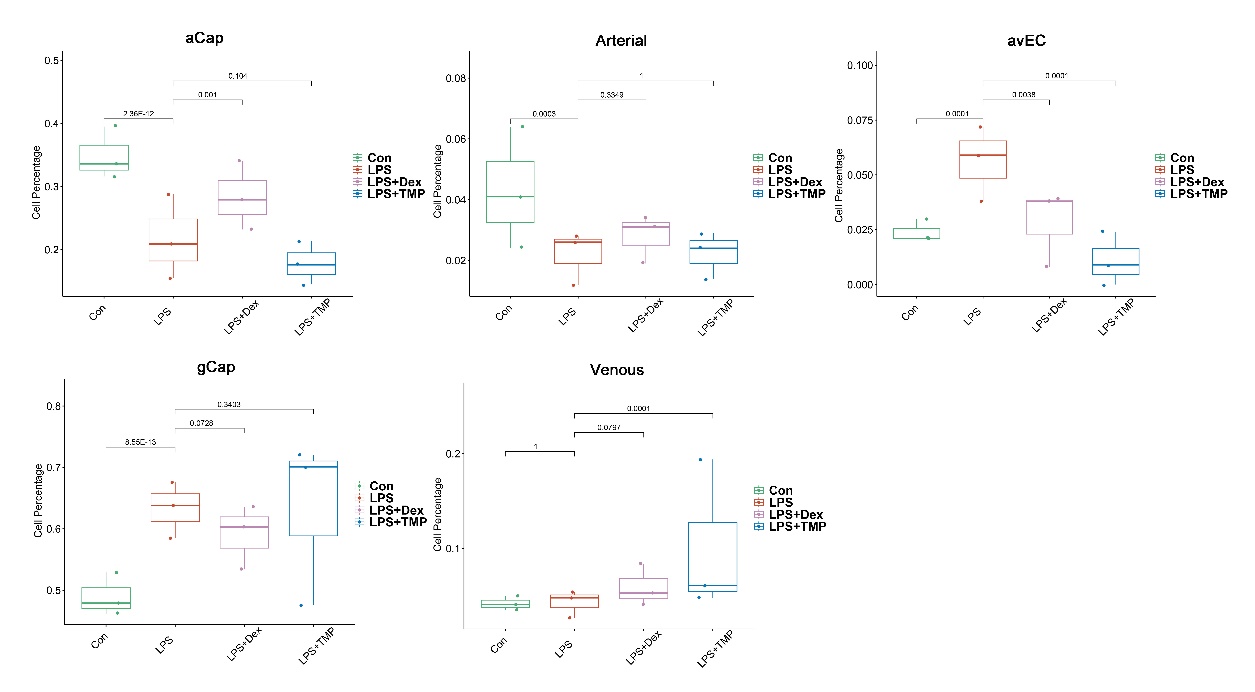


**Figure S6**

The cell percentage of endothelial cell sub-clusters as presented among the different groups. We found that LPS significantly decreased the cell percentage of Atypical Capillary (aCap) and arterial endothelial cells, whereas no significant protective effects were found after treatment with TMP. Additionally, LPS did not significantly decrease other cell subpopulations.


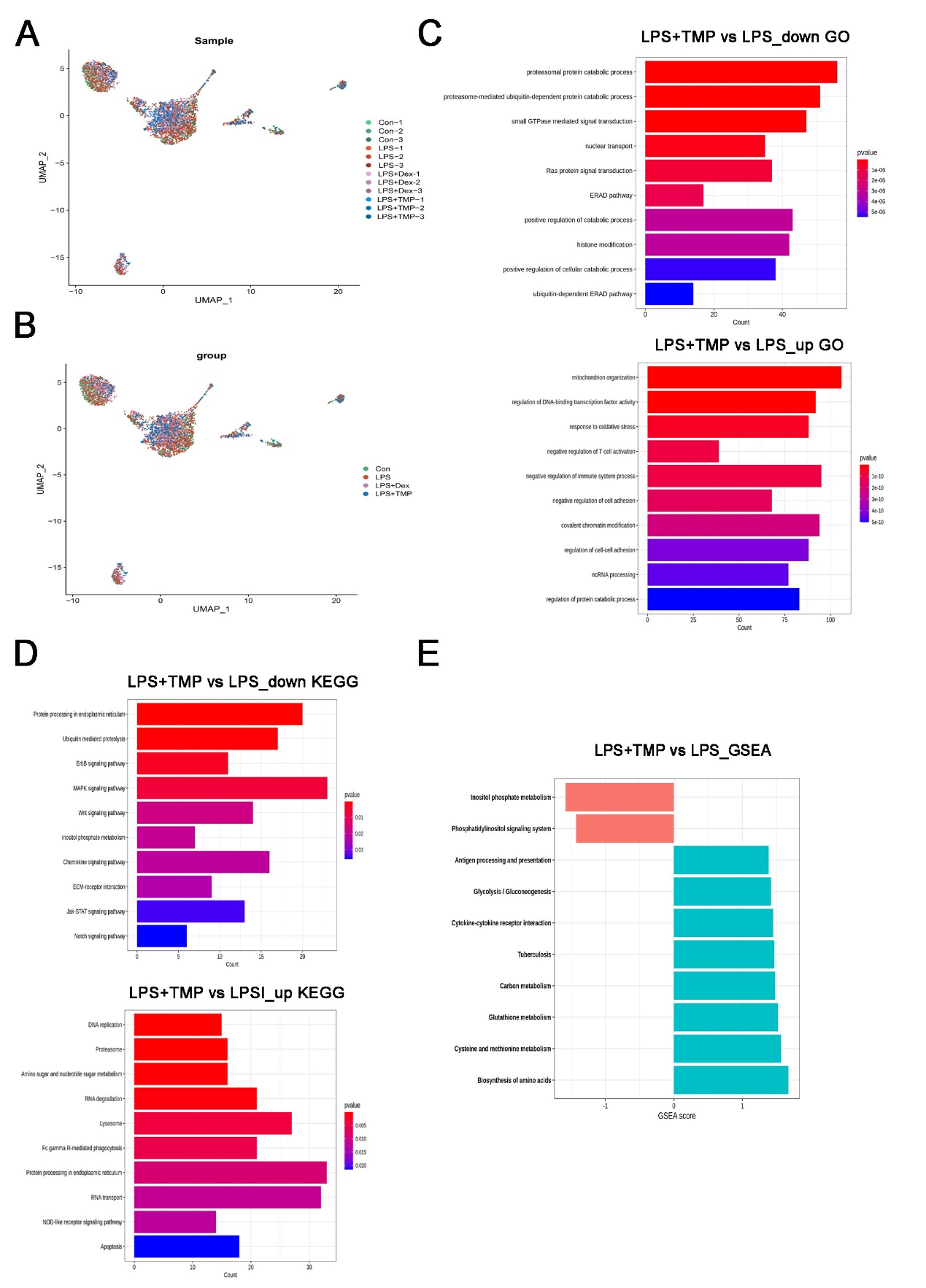


**Figure S7**

A and B: The presence of endothelial cell subsets in different samples (A) and groups (B). C and D: Enrichment analysis of the differentially expressed genes in LECs obtained after treatment with LPS and LPS+TMP using GO (C) and KEGG (D) analysis, respectively. E: GSEA analysis of the differentially expressed genes in LECs.
